# The Hidden Burden of Depression in STEMI: Effects on Early Complications and 12-Month MACCE

**DOI:** 10.3390/diagnostics16142178

**Published:** 2026-07-13

**Authors:** Alexandra Herlaș-Pop, Andrei-Flavius Radu, Ada Radu, Gabriela S. Bungau, Delia Mirela Tit, Ana Marina Marian, Victor Vlad Babeş, Elena Emilia Babes

**Affiliations:** 1 Doctoral School of Biological and Biomedical Sciences, University of Oradea, 410087 Oradea, Romania; pop.alexandra@student.uoradea.ro (A.H.-P.); gbungau@uoradea.ro (G.S.B.); dtit@uoradea.ro (D.M.T.); eebabes@uoradea.ro (E.E.B.); 2 Department of Preclinical Disciplines, Faculty of Medicine and Pharmacy, University of Oradea, 410073 Oradea, Romania; 3 Department of Psycho-Neuroscience and Recovery, Faculty of Medicine and Pharmacy, University of Oradea, 410073 Oradea, Romania; 4 Department of Pharmacy, Faculty of Medicine and Pharmacy, University of Oradea, 410028 Oradea, Romania; 5 Cardiology Department, Bihor County Clinical Emergency Hospital, 65 Gheorghe Doja Street, 410169 Oradea, Romania; marian.anamarina@student.uoradea.ro (A.M.M.); vvbabes@uoradea.ro (V.V.B.); 6 Department of Medical Disciplines, Faculty of Medicine and Pharmacy, University of Oradea, 410073 Oradea, Romania

**Keywords:** depression, ST-segment elevation myocardial infarction, systemic immune-inflammation index, major adverse cardiovascular and cerebrovascular events, C-reactive protein, left ventricular ejection fraction

## Abstract

**Background/Objectives**: Depression is a frequent but underrecognized comorbidity in ST-segment elevation myocardial infarction (STEMI) and is associated with adverse cardiovascular outcomes. The interplay between pre-existing depression, systemic inflammation, and major adverse cardiovascular and cerebrovascular events (MACCE) in STEMI remains insufficiently explored. This study aimed to investigate this interplay in a cohort of patients with STEMI. **Methods**: This retrospective observational cohort study included 292 consecutive patients with STEMI admitted between September 2023 and September 2024. Depression was identified through pre-existing psychiatric diagnoses. Inflammatory markers (C-reactive protein (CRP), neutrophil-to-lymphocyte ratio (NLR), systemic immune-inflammation index (SII)) and echocardiographic parameters, including left ventricular ejection fraction (LVEF), were collected at baseline and 12-month follow-up. In-hospital and 12-month MACCE were defined as composite endpoints. **Results**: Depression was present in 21.2% of patients. Patients with depression exhibited significantly higher CRP, NLR, SII, 48-h troponin levels, no-reflow incidence, and longer hospitalization. In-hospital MACCE occurred in 24.2% versus 11.7% of patients (*p* = 0.014), driven mainly by recurrent myocardial infarction, with a trend toward severe heart failure. At 12 months, MACCE occurred in 53.22% of patients with depression versus 23.5% of patients without (*p* < 0.001). Independent predictors of 12-month MACCE included depression (OR 3.35), baseline SII (OR 4.89 per 1000 units), baseline CRP (OR 1.48 per 10 mg/L), and 12-month LVEF (OR 0.69 per 5% increase). No significant interaction was observed between depression and inflammatory or functional parameters. **Conclusions**: Pre-existing depression was independently associated with increased in-hospital and 12-month MACCE risk in patients with STEMI. Future prospective studies should assess whether treating depression improves long-term STEMI outcomes.

## 1. Introduction

ST-segment elevation myocardial infarction (STEMI) continues to be a major contributor to global cardiovascular morbidity and mortality despite significant advances in revascularization techniques and pharmacological treatment [1]. While traditional risk stratification focuses on clinical and paraclinical markers, such as age, troponin, left ventricular ejection fraction (LVEF) and natriuretic peptides, growing evidence supports the importance of psychosocial and inflammatory factors in determining prognosis following acute coronary syndrome (ACS) [2,3,4].

Depression is a common comorbidity in patients with ACS [5] but frequently remains underrecognized and undertreated [6]. Thombs et al. [7] reported that major depression, assessed using a structured clinical interview, was diagnosed in 19.8% of patients hospitalized with acute myocardial infarction (AMI). The prevalence of clinically significant depressive symptoms varied widely, ranging from 7.3% to 31.1%, depending on the assessment questionnaire used. Consistently, a meta-analysis by Feng L. et al. found that depression was present in 28.7% of patients with AMI [8]. Symptoms of anxiety and depression are commonly reported by patients with ACS in the preceding months [9].

Depressive symptoms and/or clinical depression is associated with neurohormonal stress leading to an increased level of circulating catecholamines with up-regulation of blood pressure and heart rate, which can precipitate a cardiovascular event [7]. The importance of depression in patients with ACS has been emphasized since 2014, when the American Heart Association formally recognized depression as an independent risk factor for ACS [10].

Depression has been shown to be a cardiovascular risk factor of comparable magnitude to traditional somatic risk factors in several studies and in recent ESC guidelines [11,12,13]. Nevertheless, the management of patients with depression and ACS remains poor, with a reduced percentage receiving medication (4.1%) and psychotherapy (6.6%) [14,15]. However, the DEPACS study demonstrated that routine depression screening in patients with recent ACS and appropriate treatment of depression improved long-term cardiac outcomes [16].

Depression has been associated with poor cardiovascular outcomes [17], reduced quality of life and increased healthcare costs [18,19]. Meta-analyses indicate that depression significantly increases the risk of incident heart failure (HF), new-onset and recurrent atrial fibrillation, ventricular tachyarrhythmias, and sudden cardiac death [20,21,22,23].

Individuals meeting diagnostic criteria for major depressive disorder are therefore at particularly high risk of adverse cardiovascular events and commonly experience substantial impairment in quality of life, underscoring the need for early detection, accurate diagnosis, and targeted management [24]. Depression has been consistently linked to an increased risk of non-fatal cardiovascular events and all-cause mortality after ACS [10]. Notably, this elevated mortality risk has been shown to persist even a decade after percutaneous coronary intervention (PCI), including in predominantly elective PCI cohorts [25]. Several pathophysiologic and behavioural mechanisms are thought to be implied in the complex relationship between depression and ACS. Proposed mechanisms for the interplay between heart and brain activity include impaired adherence to treatment and lifestyle factors [26], autonomic dysregulation [27], platelet activation and endothelial dysfunction, neuroendocrine dysfunction [28] and systemic inflammation [29].

Inflammation is a key mechanism underlying atherosclerosis and myocardial injury and has been consistently shown to be an important prognostic factor in patients with ACS [30]. Inflammation is involved in cardiac repair after ACS [31], but it also contributes to plaque instability and recurrent atherothrombotic events [32,33]. Inflammatory biomarkers, including C-reactive protein (CRP) [28], interleukin-6 [34] and tumour necrosis factor-α [28], are increased in ACS and can be correlated with poor cardiac outcomes. Abnormal inflammatory responses were also observed in depression [35]. Lespérance et al. [36] demonstrated higher CRP levels in patients with depression. Although a significant interaction between CRP levels and Beck Depression Inventory scores has been reported in predicting major adverse cardiovascular events in ACS, suggesting that the effect of depression may be partly mediated through inflammatory pathways [37], other studies have found no significant differences in inflammatory status between patients with ACS who have depression and those who do not [38,39]. These conflicting findings highlight the persistent uncertainty surrounding the contribution of inflammation to the adverse cardiovascular risk associated with depression in the ACS setting.

CRP is a well-established marker of acute inflammation and has been linked to worse outcomes in ACS [28]. Recently, composite haematological indices, such as the neutrophil-to-lymphocyte ratio (NLR) and systemic immune-inflammation index (SII), have emerged as promising predictors of cardiovascular risk, capturing a broader immune response through neutrophil, platelet, and lymphocyte counts [40,41,42].

Depression itself is associated with elevated NLR and SII, independent of cardiac disease. Demir et al. [43] found that patients with major depressive disorder had significantly higher NLR than controls. An elevated SII was reported in patients with moderate/major depressive disorder in recent studies [44,45]. This supports the hypothesis that patients with STEMI who have depression may exhibit higher NLR and SII, reflecting an additive inflammatory burden.

Despite the extensive literature on both depression and inflammation as individual risk factors, few studies have explored their combined impact on long-term outcomes in patients with STEMI [46,47]. Moreover, the potential interaction between psychological and biological stress responses remains underexplored. Understanding how depression and inflammation jointly influence prognosis could improve risk stratification and guide more personalized secondary prevention strategies. Inflammatory marker levels vary over time across different phases of ACS and fluctuate with depression status; therefore, studies assessing inflammation and depression at a single time point may fail to capture their true causal relationship. Most existing studies focus on short-term outcomes, but the long-term interaction between depression and inflammation is less explored. Since many complications, including recurrent MI, HF progression, stroke, or death, occur after discharge and depression is a chronic condition with long-term effects, it is important to assess the impact of depression and inflammation on long-term MACCE.

The aim of this study was to investigate the association between baseline depression, inflammatory markers (CRP and SII), and major adverse cardiovascular and cerebrovascular events (MACCE) both during hospitalization and at 12-month follow-up in patients with STEMI. In addition to baseline values, we evaluated the evolution of key inflammatory and cardiac parameters over time, including changes in CRP, SII and LVEF, to assess their predictive value for long-term outcomes. We also explored potential interactions between depression, inflammation, and cardiac function in predicting MACCE to better understand whether these factors act independently or synergistically in shaping post-STEMI prognosis.

## 2. Materials and Methods

### 2.1. Study Design and Population

This retrospective, observational cohort study included consecutive patients admitted with STEMI to the Cardiology Department of Bihor County Emergency Clinical Hospital between September 2023 and September 2024. STEMI was defined according to contemporary ESC guidelines based on clinical symptoms, ECG criteria, and biomarker elevation [1]. The study was conducted in accordance with the Declaration of Helsinki and was approved by the Bihor County Emergency Clinical Hospital Ethics Committee (Approval No. 14912/15.05.2025).

**Figure 1:**
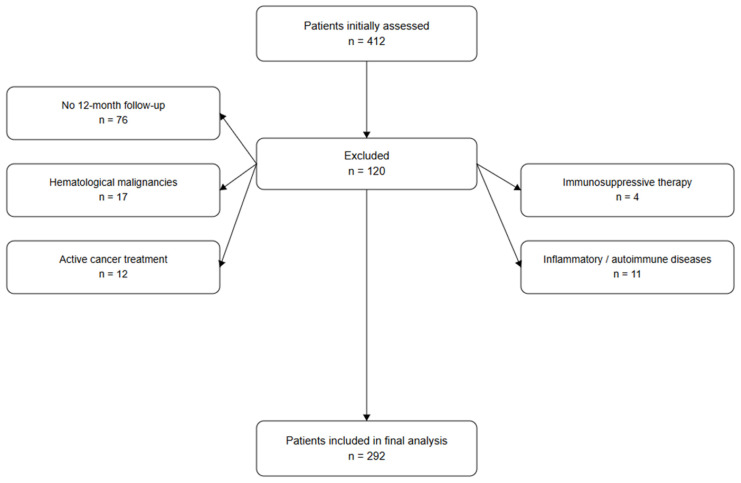
Study flowchart.

Patients were included if they had documented clinical data at index hospitalization and complete follow-up records up to 12 months after the event. Of the 412 patients initially screened, 76 were excluded due to missing 12-month follow-up data. Additional exclusions included patients with known haematological malignancies (*n* = 17), active cancer treatment (*n* = 12), immunosuppressive therapy (*n* = 4), and concomitant diagnosed inflammatory/autoimmune diseases (*n* = 11) (Figure 1).

Following the application of the inclusion and exclusion criteria, a total of 292 patients were further analysed. Considering the retrospective nature of the study, the sample size was established based on the total number of eligible patients admitted throughout the study period. This approach facilitated a thorough assessment of the real-world clinical data. A post hoc power analysis was conducted for the final multivariable logistic regression models. For the in-hospital MACCE model, which included 292 patients with STEMI and 42 events with 5 predictors, the study had approximately 80–85% power to detect an odds ratio ≥ 2.0 at a significance level of 0.05. Similarly, the 12-month MACCE model included 87 events and 4 predictors and was analysed using Firth’s penalized logistic regression. This approach ensured stable coefficient estimation and sufficient power (>90%) to detect moderate effect sizes while minimizing small-sample bias.

### 2.2. Assessment of Depression

Depression status was identified retrospectively based on pre-existing psychiatric diagnoses documented in the electronic medical record. Only patients with a diagnosis established by a psychiatrist prior to the index STEMI were classified as having depression. Because this was a retrospective analysis, standardized assessments of depressive symptoms (e.g., PHQ-9, BDI, or HADS), symptom severity scores, timing of depressive episodes, and details regarding antidepressant adherence were not consistently available and therefore were not included in the analysis. Consequently, the study evaluated the prognostic impact of clinically documented pre-existing depression rather than depressive symptom burden at the time of STEMI presentation, and cases of previously unrecognized or post-MI reactive depression could not be identified.

### 2.3. Clinical, Laboratory, and Echocardiographic Data Collection

Baseline demographic characteristics, cardiovascular risk factors, comorbidities, Killip class, procedural characteristics, in-hospital angiographic findings, 12-month complications, and length of hospital stay were collected. The no-reflow phenomenon was defined as inadequate myocardial reperfusion despite successful epicardial coronary artery opening, as indicated by a final Thrombolysis in Myocardial Infarction (TIMI) flow grade < 3 and/or a myocardial blush grade (MBG) < 2.

Laboratory parameters recorded from medical files at index hospitalization included complete blood count (CBC), CRP, and cardiac biomarkers: Hs-Tni (baseline and at 48 h after admission), N-terminal pro-B-type natriuretic peptide (NT proBNP), lipid profile, and creatinine level. The SII was calculated as follows: SII = platelets × neutrophils/lymphocytes. Laboratory parameters, CBC, NT-proBNP, lipid profile, and CRP, were also analysed from medical data recorded at the 1-year assessment. CBC analyses were performed using the Alinity hq automated haematology analyser from Abbott, Abbott Park, IL, USA. This advanced device employs Multi-Angle Polarized Scatter Separation technology, allowing for effective differentiation of white blood cells, alongside laser light scatter techniques for assessing red blood cells and platelets. Hs-Tni levels were measured using the Abbott Alinity hs-Tni assay, while CRP concentrations were quantified on the same Abbott Alinity platform through a turbidimetric assay.

Echocardiographic examinations performed during hospitalization and at 12 months were reviewed. LVEF was measured using the Simpson biplane method. ΔLVEF was defined as the absolute change between discharge and 12-month follow-up. Echocardiography was performed on a Vivid E 95 (GE Vingmed Ultrasound, Horten, Norway) or on a Philips CX50 point-of-care ultrasound device (Philips Healthcare, Taguig, Philippines).

Coronary angiography (CAG) was performed via a radial artery approach, and before the procedure, all patients received 300 mg of acetylsalicylic acid, a loading dose of a P2Y12 inhibitor, and 70–100 U/kg of unfractionated heparin. Angiographic images were independently reviewed by two experienced cardiologists who were blinded to the study data. Following CAG, all patients were treated with standard therapy, including dual antiplatelet agents (aspirin with either clopidogrel or ticagrelor). Additional medications, such as beta-blockers, ACE inhibitors or angiotensin receptor blockers, and high-intensity statins, were prescribed at the discretion of the attending physician.

Because of the retrospective design, treatment adherence, lifestyle factors, and outpatient management could not be assessed.

### 2.4. Outcomes

In-hospital MACCE was defined as a composite of all-cause mortality, resuscitated cardiac arrest, reinfarction, stroke, severe HF (Killip class ≥ III), and repeated unplanned revascularization.

#### Definitions of MACCE Components

Cardiac death was defined as death due to cardiac causes, including pump failure, arrhythmia, reinfarction, mechanical complications, or cardiac arrest, based on clinical documentation and adjudication.

Recurrent MI was defined as an AMI occurring after the initial ACS event, characterized by the return of chest pain accompanied by new ST-segment elevation or the development of new pathological Q waves in at least two contiguous leads, along with a rise in hs-troponin measured using two samples taken 3–6 h apart. If the initial hs-troponin level had already fallen to the normal range, standard diagnostic criteria for a new acute MI were applied. Conversely, when the initial hs-troponin level remained elevated but was on a downward trend, the diagnosis of recurrent MI required a subsequent increase of more than 20% in hs-troponin [3].

Stroke or transient ischaemic attack (TIA) was defined by clinical diagnosis and confirmed by neuroimaging (CT or MRI) and neurologist assessment.

Repeat revascularization was defined as any unplanned revascularization procedure performed after the initial PCI due to recurrent ischaemic symptoms or a recurrent MI. This category included the following: target lesion revascularization, defined as repeat PCI for restenosis at the originally treated lesion or within 5 mm of it; target vessel revascularization, defined as unplanned PCI for a new stenosis elsewhere within the same coronary artery treated during the initial PCI; and another unplanned vessel revascularization, defined as PCI involving a coronary artery different from the one treated initially. Planned PCI procedures intended to complete revascularization were classified as staged revascularizations and were not considered repeat revascularizations [48].

HF was defined based on clinical evaluation combined with data from transthoracic echocardiography, performed within the first 24 h after patient admission. Only patients in Killip classes III and IV were counted as having MACCE.

The objective of the present study was to evaluate the cumulative burden of adverse cardiovascular events from the time of STEMI presentation through 12 months of follow-up. Therefore, for the 12-month MACCE analysis, we included all the above in-hospital events that occurred during the index admission and during the 12-month follow-up period. This approach is consistent with the standard definition of composite cardiovascular endpoints, where early complications are considered part of the overall trajectory of post-MI risk. Patients who experience severe in-hospital events remain at high risk for subsequent adverse outcomes or may not survive to discharge excluding these events, but the true burden of morbidity and mortality associated with STEMI is often underestimated. Therefore, both events occurring during hospitalization and those recorded after discharge were incorporated into the composite 12-month MACCE endpoint to ensure a comprehensive and clinically meaningful assessment of patient outcomes. The 1-year MACCE outcome was defined as a composite of all-cause death, MI, stroke, repeated unplanned revascularization, severe HF requiring hospital admission, or resuscitated cardiac arrest occurring at any time from admission through the 12-month follow-up.

To specifically evaluate post-discharge prognosis, a landmark analysis was performed including only patients discharged alive after the index STEMI.

Event data were obtained from hospitalization records, outpatient documentation, and digital follow-up records within the institutional electronic medical record system. Multiple events occurring in the same patient were recorded only once.

### 2.5. Statistical Analysis

Continuous variables are presented as mean ± standard deviation and categorical variables as counts and percentages. Group comparisons were performed using Student’s *t*-test or the Mann–Whitney U test for continuous variables and the χ^2^ or Fisher’s exact test for categorical variables. Multivariable logistic regression was used to identify independent predictors of in-hospital and 12-month MACCE, with variables selected based on clinical relevance and univariable associations. Continuous variables were scaled to clinically meaningful units to improve interpretability and model stability (CRP per 10 mg/L, SII, hs-troponin and NT-proBNP per 1000 units, and LVEF per 5% increase).

Standard multivariable logistic regression was applied for in-hospital MACCE. For the 12-month analysis, Firth penalized logistic regression was used to reduce potential bias in maximum likelihood estimation and improve coefficient stability in the final parsimonious multivariable model. Conventional logistic regression using the same final predictor set was additionally performed as a sensitivity analysis. Biologically plausible interaction terms (e.g., Depression × CRP, Depression × SII, Depression × LVEF) and models incorporating change (Δ) variables were tested to assess whether worsening inflammation or cardiac dysfunction differentially affected outcomes in patients with depression. Time-to-event analyses were performed using Kaplan–Meier curves and the log-rank test. Model discrimination was assessed using the area under the receiver operating characteristic curve (AUC), whereas calibration of the conventional logistic regression model was evaluated using the Hosmer–Lemeshow goodness-of-fit test. Statistical analyses were conducted using SPSS version 25 (IBM Corp., Armonk, NY, USA) and R Core Team, (Vienna, Austria) version 4.5.1 (2025), with a two-sided *p*-value < 0.05 considered statistically significant.

## 3. Results

### 3.1. Baseline Characteristics of Patients with STEMI

There were 292 patients with STEMI enrolled in this study, 62 of them (21.2%) with a pre-existing diagnosis of depression. The baseline clinical and paraclinical characteristics are revealed in Table 1.

The mean age was comparable between patients with and without depression (*p* = 0.55). There were no significant differences in body mass index (BMI), smoking status, hypertension, diabetes, or lipid profiles between the groups (all *p* > 0.20). Patients with STEMI with a pre-existing diagnosis of depression were more commonly females (*p* < 0.001) and had a higher grade of inflammation (increased CRP (*p* = 0.001), NLR (*p* = 0.001) and SII (*p* = 0.012)). Furthermore, these patients had a significantly higher hs-troponin level at 48 h (*p* = 0.035) and experienced the no-reflow phenomenon more often (*p* < 0.001). The length of hospital stay was longer in patients with STEMI with depression (*p* = 0.019). There were no significant differences in haemoglobin, creatinine, NT-proBNP, or LVEF between the groups. The extent of coronary artery disease, as assessed by the number of affected vessels, was similar.

### 3.2. In-Hospital MACCE in Patients with STEMI

During hospitalisation, there were 42 patients (14.32%) who developed MACCE. It appeared more commonly in the group of patients with pre-existing depression (*p* = 0.014). In-hospital MACCE in patients with STEMI with and without depression are presented in Table 2.

Recurrent MI was significantly more frequent (*p* = 0.03), and severe heart failure had a trend towards significance (*p* = 0.06). All the other components of MACCE were more frequent in the depression group although they did not reach statistical significance.

Patients with STEMI who developed in-hospital MACCE were compared with patients without MACCE regarding their baseline characteristics. The results are presented in Table 3.

In-hospital MACCE occurred in 14.3% of patients with STEMI and was significantly associated with older age, higher levels of inflammatory markers (CRP, WBC, and SII), renal dysfunction, elevated cardiac injury biomarkers (troponin and NT-proBNP), and cholesterol. Depression was also significantly more prevalent among patients who experienced MACCE. These findings suggest a multifactorial risk profile for early in-hospital complications, with contributions from inflammatory burden and psychological comorbidity.

A multivariable logistic regression model was constructed to identify independent predictors of in-hospital MACCE, including depression, SII, CRP, LVEF, age, troponin, cholesterol, and NT-proBNP (variables with *p* < 0.05 in univariate analysis). These biomarkers reflect myocardial injury severity, hemodynamic stress, and systemic inflammatory activation, which are central mechanisms underlying early post-STEMI complications. Model calibration was satisfactory across all variants (Hosmer–Lemeshow *p* = 0.58–0.72), with overall classification accuracy ranging from 85.5% to 86.6%. The final in-hospital MACCE logistic regression model demonstrated good discrimination, with an area under the receiver operating characteristic curve (AUC) of 0.828 (95% CI 0.761–0.896).

**Figure 2:**
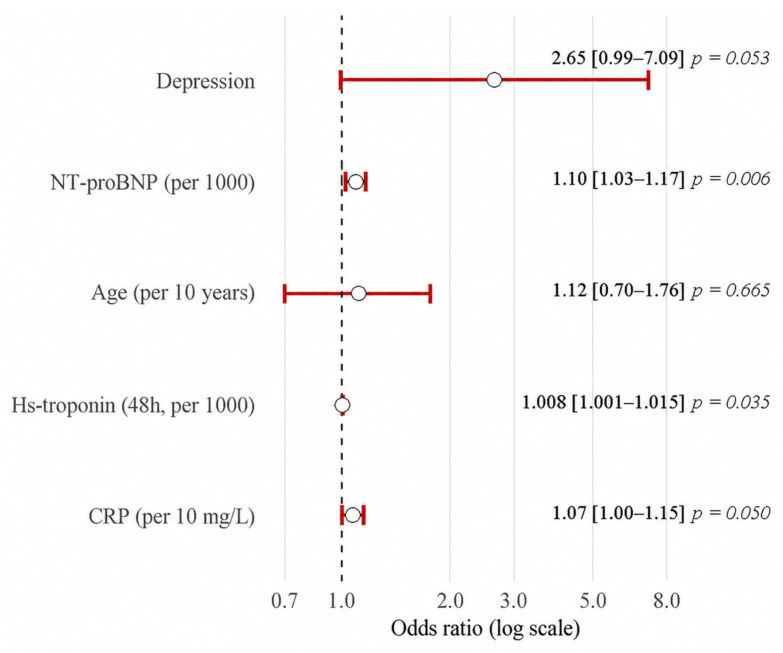
Predictors of in-hospital MACCE. Forest plot showing independent predictors of in-hospital major adverse cardiac and cerebrovascular events (MACCE). Odds ratios (ORs) and 95% confidence intervals (CI) are displayed on a logarithmic scale. Red markers indicate harmful associations (OR > 1). Continuous predictors were expressed per clinically meaningful increments (e.g., age per 10 years, CRP per 10 mg/L, NT-proBNP and troponin per 1000 units) to facilitate interpretation of ORs. The vertical dashed line represents the null effect (OR = 1).

NT-proBNP (OR ≈ 1.10 per 1000 units; *p* = 0.005–0.006) and hs-troponin (OR ≈ 1.008–1.009 per 1000 units; *p* = 0.020–0.035) emerged as consistent and independent predictors of in-hospital MACCE. In contrast, baseline SII, LVEF, cholesterol, and age were not independently associated with early adverse events. The final reduced model (a maximum of five predictors (10 events per variable) identified NT-proBNP and troponin as the primary independent predictors of in-hospital adverse events, with weak trends observed for depression and inflammation (Table 4, Figure 2).

Depression demonstrated a borderline association with in-hospital MACCE (OR ≈ 2.6; *p* = 0.053–0.065), and CRP showed a similar trend (*p* = 0.05–0.10). These findings suggest that while acute cardiac injury and hemodynamic stress are the primary drivers of early complications, psychological vulnerability and inflammatory activation may contribute additively to short-term risk. The absence of an independent association for SII in the acute phase suggests that systemic inflammation may exert a more prominent influence on longer-term outcomes rather than immediate post-infarction events.

Given prior evidence suggesting a biological link between depression and systemic inflammation in cardiovascular disease, we conducted an exploratory analysis to assess whether the effect of depression on MACCE was modified by CRP levels. To test this hypothesis, we introduced an interaction term (Depression × CRP) into a second regression model, evaluating whether inflammation mediated or amplified the impact of depression on short-term outcomes. To preserve model parsimony and minimize overfitting, only variables significantly associated with in-hospital MACCE in the initial multivariable analysis were retained in the interaction model. The model included depression, CRP, their interaction term and NT-proBNP. Other covariates were excluded to maintain an acceptable events-per-variable ratio for statistical validity.

The interaction term (Depression × CRP) showed a non-significant moderation effect of inflammation on the relationship between depression and adverse outcomes (*p* = 0.09, OR = 0.99, 95% CI 0.975–1.002). These findings support the additive contribution of psychological and inflammatory risk factors in the acute setting.

As a sensitivity analysis, we evaluated the potential confounding effect of the no-reflow phenomenon by replacing CRP with no reflow in the multivariable logistic regression model while maintaining the same number of predictors to avoid model overfitting. Depression remained independently associated with in-hospital MACCE (OR 3.51, 95% CI 1.34–9.16; *p* = 0.010), whereas no reflow was not independently associated with the outcome after adjustment for NT-proBNP and hs-troponin (OR 0.87, 95% CI 0.29–2.61; *p* = 0.799). NT-proBNP and hs-troponin remained significant independent predictors, supporting the robustness of the primary findings.

An exploratory depression × sex interaction analysis was performed to evaluate whether the prognostic impact of depression differed between women and men. No significant interaction was observed (OR 0.99, 95% CI 0.16–6.12; *p* = 0.992), indicating no evidence of effect modification by sex.

**Figure 3:**
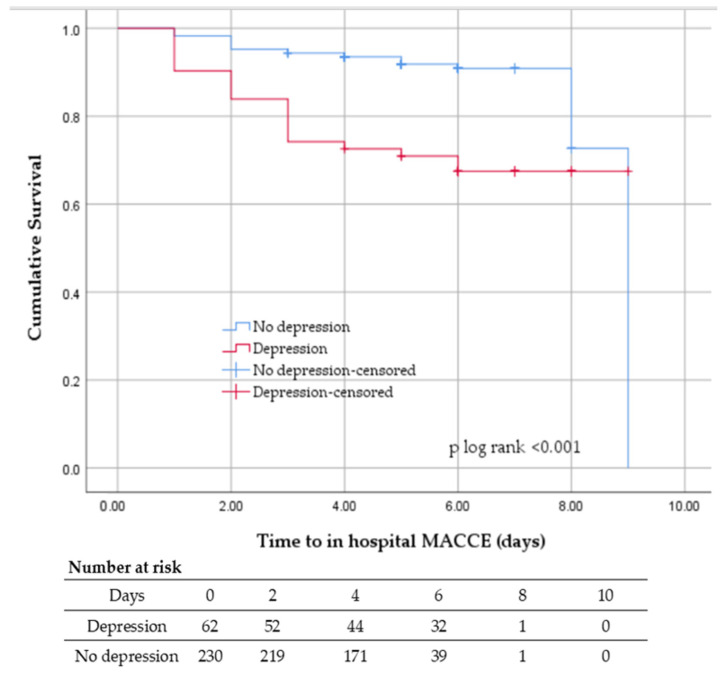
Kaplan–Meier survival curve free of in-hospital MACCE. The numbers below the plot indicate the number of patients at risk at each time point. The log-rank test was used to compare survival distributions.

The Kaplan–Meier curve for in-hospital MACCE shows an early and pronounced separation between patients with and without depression (Figure 3). Individuals with depression experienced a significantly higher rate of in-hospital complications, with events occurring sooner during the hospitalization period. By day 10, the cumulative MACCE-free survival remained substantially lower among patients with depression compared with the non-depressed group. This difference emerged within the first 1–2 days after admission, emphasizing that the presence of depression may be associated with greater clinical vulnerability during the acute phase of MI. The log-rank test confirmed the statistical significance of this divergence (*p* < 0.001). Given the short duration of in-hospital follow-up, these findings should be interpreted cautiously and primarily illustrate the temporal distribution of early in-hospital events.

### 3.3. Twelve-Month MACCE in Patients with STEMI

To assess the extended prognostic value of depression and systemic inflammation, we evaluated 12-month MACCE in this cohort. For the 12-month analysis, all patients admitted with STEMI were included regardless of whether they experienced in-hospital events or death. Predictors included baseline parameters, values measured at 1 year, and the absolute change (Δ) between the two time points. In the first year after STEMI, MACCE occurred among 87 (29.79%) patients. Patients in the depression group had significantly more MACCE (53.32%) than patients without depression (23.48%). Twelve-month MACCE in patients with STEMI with and without pre-existing depression is depicted in Table 5.

Depression at baseline remains strongly associated with MACCE at 12 months, especially with recurrent MI and severe HF requiring hospitalization. Overall, the MACCE rate was nearly double in patients with depression. The baseline parameters in patients with STEMI with and without 12-month MACCE are presented in Table 6.

MACCE at 1-year was significantly more common in patients with depression (*p* < 0.001). An increased baseline CRP and SII was also found in patients who experienced long-term MACCE. Furthermore, baseline hs-troponin (48 h) and NT-proBNP were significantly increased in patients with 12-month MACCE. Baseline LVEF was significantly lower in patients who developed long-term MACCE. The comparison of 12-month characteristics in patients with and without long-term MACCE is presented in Table 7.

Patients who experienced MACCE within 1 year exhibited higher levels of inflammatory markers (CRP and SII) and showed a smaller reduction in CRP and SII (ΔCRP and ΔSII) over the 12-month follow-up. LVEF was significantly lower, and the improvement in LVEF over 12 months (ΔLVEF) was significantly attenuated in the MACCE group. Patients with MACCE also had a 12-month lower HDL-cholesterol level and a higher triglycerides level.

Depression was associated with higher levels of CRP (27.51 ± 40.48 vs. 16.20 ± 31.94, *p* = 0.045) and SII (1053.10 ± 1098.42 vs. 825.193 ± 1084.50, *p* = 0.2) at 12 months compared with non-depression, reflecting persisting chronic inflammation.

The primary objective was to evaluate whether depression independently predicted 12-month MACCE and whether its effect was modified by inflammatory or cardiac functional parameters. Univariate analyses identified several predictors associated with MACCE, including depression, baseline SII, CRP, NT-proBNP, hs-troponin (48 h) and LVEF, ΔLVEF, and 12-month LVEF, ΔCRP, HDL cholesterol, and triglycerides. An initial conventional multivariable logistic regression model including candidate predictors selected based on univariate associations and clinical relevance produced unstable coefficient estimates, characterized by inflated effect sizes and wide confidence intervals for several variables, reflecting sparse data and the limited number of outcome events. To improve model stability while preserving clinical interpretability, correlated predictors were reduced based on clinical relevance and biological plausibility. The resulting parsimonious model included pre-existing depression, baseline SII, baseline CRP, and 12-month LVEF and was subsequently analysed using Firth penalized logistic regression.

**Figure 4:**
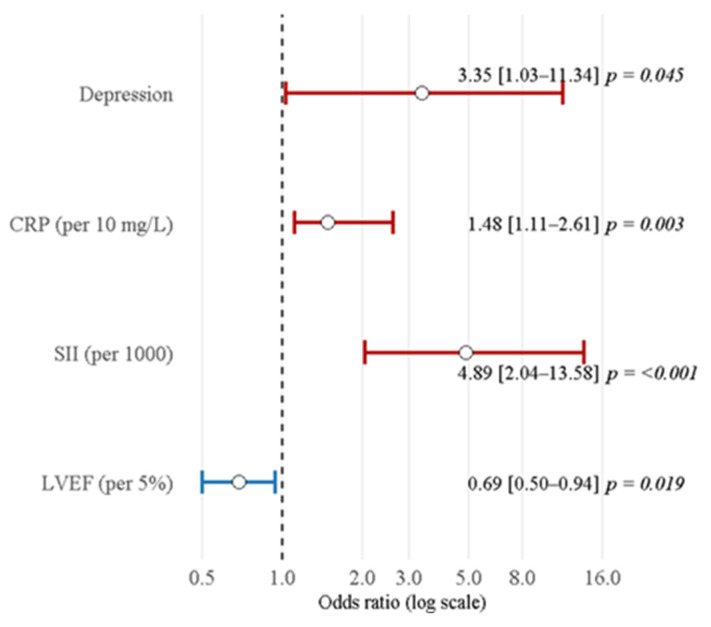
Predictors of 12-month MACCE. Forest plot of Firth penalized logistic regression results for 12-month MACCE. Odds ratios (ORs) with 95% confidence intervals are shown on a logarithmic scale. Red denotes harmful associations, and blue denotes protective associations. The dashed vertical line indicates OR = 1. ORs are expressed per predefined increments for continuous variables. ORs are reported per clinically meaningful increments (CRP per 10 mg/L, SII per 1000 units, and LVEF per 5%).

In Firth penalized logistic regression analysis, pre-existing depression was independently associated with a higher risk of 12-month MACCE (*p* = 0.045). Inflammatory markers were also significant predictors, with both CRP (per 10 mg/L increase; *p* = 0.003) and SII (per 1000 units, *p* < 0.001) showing strong associations. Conversely, higher LVEF was protective, with each 5% increase associated with a 31% reduction in MACCE risk (*p* = 0.019) (Table 8, Figure 4). The model demonstrated good discrimination, with an area under the receiver operating characteristic curve (AUC) of 0.775 (95% CI 0.688–0.863).

As a sensitivity analysis, the same parsimonious predictor set was fitted using conventional multivariable logistic regression. The results were broadly consistent with those obtained using Firth penalized logistic regression. Pre-existing depression remained independently associated with 12-month MACCE (OR 3.40, 95% CI 1.10–15.31; *p* = 0.048), while baseline SII (OR 2.15, 95% CI 1.46–3.16; *p* < 0.001) and 12-month LVEF (OR 0.42, 95% CI 0.33–0.53; *p* < 0.001) also remained significant independent predictors. Baseline CRP was not independently associated with the outcome (OR 1.01, 95% CI 0.95–1.09; *p* = 0.71). Overall, these findings support the stability of the primary Firth penalized regression model.

To specifically evaluate post-discharge prognosis, a landmark analysis was performed including only patients discharged alive after the index STEMI. In this Firth penalized logistic regression model, baseline SII (OR 2.33, 95% CI 1.60–3.62; *p* < 0.001), baseline CRP (OR 1.02, 95% CI 1.00–1.04; *p* = 0.032), and 12-month LVEF (OR 0.47, 95% CI 0.36–0.58; *p* < 0.001) remained independently associated with post-discharge MACCE. Pre-existing depression also demonstrated a borderline independent association with post-discharge events (OR 2.35, 95% CI 0.98–5.59; *p* = 0.053).

To explore the robustness of the main findings and to assess the prognostic relevance of inflammatory dynamics, a sensitivity analysis was performed using Firth penalized logistic regression models in which baseline CRP was replaced by ΔCRP. In alternative Firth penalized regression models incorporating ΔCRP as a marker of inflammatory dynamics, ΔCRP did not retain independent prognostic significance (OR 1.02 per 10 mg/L increase, 95% CI 0.94–1.10, *p* = 0.65). The association between pre-existing depression and 12-month MACCE remained of similar magnitude but reached only borderline statistical significance (OR 3.46, 95% CI 0.97–12.68, *p* = 0.057). This suggests that depression’s prognostic impact is robust, but the loss of statistical significance reflects fewer effective degrees of freedom and higher uncertainty when ΔCRP replaces baseline CRP. In contrast, SII and LVEF remained strong and independent predictors across all models. This reinforces that immune activation, not CRP change, is the dominant inflammatory pathway associated with long-term events. These findings indicate that the adverse prognostic impact of depression is robust and not mediated by changes in CRP, supporting an independent and additive contribution to long-term risk.

Independent predictors of 12-Month MACCE were depression, baseline SII, CRP and 1-year LVEF. Pre-existing depression independently triples the risk of 12-month MACCE even after adjusting for inflammation and cardiac function. Inflammation is a strong driver of risk, with SII being a particularly powerful predictor, reflecting systemic immune activation. A higher systemic inflammatory index at baseline significantly increased long-term risk. SII reflects neutrophil-mediated inflammation, platelet activation, and lymphocyte suppression, components known to aggravate cardiovascular instability and remodelling. Higher LVEF at 12 months was independently protective. Every 5% increase in LVEF reduces MACCE risk by ~31%. This confirms that the degree of ventricular recovery strongly influences prognosis. ΔLVEF was not significant. Age did not improve the model; it acted as a negative confounder and inflated depression ORs without making a meaningful contribution. Troponin and NT-proBNP were associated with in-hospital MACCE but not long-term MACCE and worsened the calibration of the 12-month model.

To investigate whether depression exerted its effect through interaction with cardiac function or inflammation, interaction models were tested: Depression × CRP, Depression × 1 year-LVEF, Depression × ΔCRP and Depression x SII. All interaction terms were non-significant (*p* = 0.27–0.81). This indicated that the effect of depression was additive, not synergistic, with inflammatory or cardiac markers.

**Figure 5:**
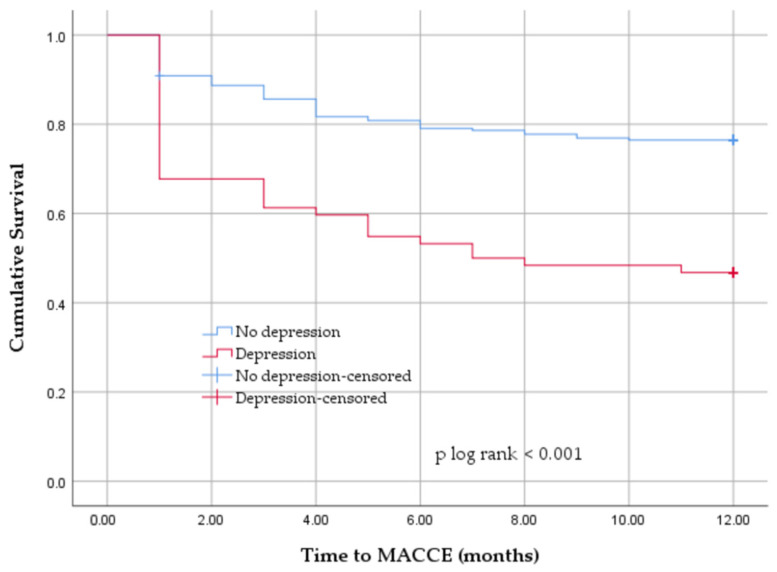
Kaplan–Meier survival curve free of 12-month MACCE.

Kaplan–Meier survival analysis demonstrated a clear and early divergence in MACCE-free survival between patients with and without depression (Figure 5). Individuals with depression experienced significantly more events during the 12-month follow-up period, with the separation between the curves becoming evident within the first months after discharge and widening progressively thereafter. By one year, the cumulative event-free survival remained markedly lower in the depression group, reflecting both a higher incidence and earlier occurrence of MACCE. The log-rank test confirmed that this difference was statistically significant (*p* < 0.001). Collectively, these findings suggest that depression is not only relevant for long-term prognosis but also confers an adverse impact during the initial index MI hospitalization.

## 4. Discussion

In this retrospective cohort study of patients with STEMI, we evaluated the prognostic significance of baseline depression and inflammatory markers on both in-hospital and 12-month MACCE.

### 4.1. Depression and Inflammation in Patients with STEMI

The prevalence of a pre-existing diagnosis of depression in our cohort of patients with STEMI was 21.2%. Similar results were reported by Thombs et al., who found that prevalent depression (existing before MI) was present in about 20% of patients with MI, including those with STEMI [15]. The ENRICHD Trial showed that major or minor depression was present in 20% of patients post-MI, with a significant proportion being previously diagnosed [49]. A meta-analysis including 25 studies reported a pooled prevalence of depression after AMI of 23.58% (95% CI: 22.86–24.32%) [50]. A higher prevalence of depression is reported in women and younger patients with STEMI. Similarly, in our cohort, women were more commonly affected by pre-existing depression. Depression is often underdiagnosed in men and older patients, suggesting that the real prevalence might be underestimated. Depression may be underreported in patients with heart disease due to stigma, lack of routine screening, and overlap with cardiac symptoms (e.g., fatigue, sleep disturbance) [13].

Depression was more frequent among women than men in our cohort, consistent with previous epidemiological studies showing an approximately two-fold higher prevalence of depression in women. Beyond psychosocial factors, several biological mechanisms may contribute to the increased cardiovascular vulnerability of women with depression. A decline in oestrogen levels after menopause is associated with endothelial dysfunction, oxidative stress, vascular inflammation, and impaired coronary microvascular function. Increased sympathetic nervous system activation and hypothalamic–pituitary–adrenal axis responses to psychological stress are also more common in women, potentially enhancing platelet activation and inflammatory signalling. Furthermore, smaller coronary artery dimensions and a higher prevalence of coronary microvascular dysfunction may exacerbate myocardial ischaemia despite successful epicardial reperfusion. These mechanisms may provide a biological explanation for the association between depression and adverse cardiovascular outcomes in women following STEMI [51]. However, our exploratory depression × sex interaction analysis did not demonstrate significant effect modification, and the study was not sufficiently powered for sex-stratified analyses.

In our study, patients with pre-existing depression who experienced STEMI had significantly higher levels of baseline inflammation, as reflected by CRP, NLR and SII. These findings are statistically and clinically significant even though traditional cardiovascular risk factors were similar between groups. Routine screening for depression in patients with STEMI may help identify those with an exaggerated inflammatory response. These patients may benefit from closer monitoring, targeted anti-inflammatory interventions, or integrated psychiatric support.

Furthermore, patients with depression had a higher incidence of no reflow and elevated troponin levels at 48 h, indicating more severe myocardial injury. The percentage of patients experiencing no reflow after PCI for STEMI varies across studies but is generally reported in the range of 5% to 30% of patients with STEMI undergoing primary PCI. In STEMI registries like Euro Heart, no reflow was reported in 10–25% of patients [52], and a recent study revealed an incidence of 25% in patients with STEMI undergoing primary PCI [53]. No reflow is a “multifactorial phenomenon” in ACS/PCI, with systemic inflammation, distal embolization, reperfusion injury, and microvascular dysfunction as major mechanisms [54,55]. Patients with depression experienced no reflow more frequently in our study (30.65% vs. 10.43%), probably due to microvascular dysfunction and increased inflammation, which are well-recognized contributors to the no-reflow phenomenon in STEMI treated with primary PCI. Although our cohort showed a higher rate of no reflow among patients with depression, we found no direct prior evidence in the literature linking depression per se to no reflow. Given the established relationship between depression and systemic inflammation, our finding is hypothesis-generating, supporting further research into psychological factors as potential risk markers for microvascular reperfusion failure. The association between depression and the no-reflow phenomenon seems to be indirect and mediated through shared pathophysiological mechanisms. Depression is linked to autonomic nervous system imbalance with increased sympathetic activity, endothelial dysfunction with reduced nitric oxide availability, chronic low-grade inflammation, and heightened platelet activation. Together, these processes promote coronary microvascular impairment, distal embolization, and reperfusion injury, all of which contribute to the development of no reflow during PCI [47,56]. Although patients with depression experienced the no-reflow phenomenon more frequently, sensitivity analysis demonstrated that adjustment for no reflow did not materially alter the association between depression and in-hospital MACCE. These findings suggest that depression provides prognostic information beyond acute procedural complications and microvascular reperfusion failure.

### 4.2. Depression, Inflammation and In-Hospital MACCE

In-hospital MACCE occurred in 14.8% of our cohort, with all-cause death and cardiac death responsible for 3.76% and 3.42% of cases, respectively. Similar data are revealed in contemporary registries, with in-hospital mortality after STEMI in the primary PCI period of around 4–8% and cardiac death being the dominant component [57,58]. In-hospital reinfarction, stroke, and urgent repeat revascularization occur less frequently (typically each in the 1–3% range), whereas severe HF or cardiogenic shock and resuscitated cardiac arrest are reported in about 5–10% and 2–5% of patients, respectively, depending on case mix and definitions [57]. Similar results were observed in our cohort, where reinfarction and repeated unplanned myocardial revascularisation occurred in 1–2% of cases, while resuscitated cardiac arrest was observed in 7.53% of cases and severe HF was reported in 8.21% of cases.

Depression was significantly associated with higher in-hospital MACCE, driven in part by recurrent MI and severe HF. Mortality, resuscitated cardiac arrest, repeated unplanned revascularisation and stroke were numerically higher but not statistically significant in patients with depression. CRP, NLR, SII, and troponin (48 h) were all significantly higher in patients who developed in-hospital MACCE. LVEF was lower and NT-proBNP was higher in patients with MACCE. These findings support the role of inflammation and myocardial dysfunction in adverse outcomes. In multiple regression analysis, NT-proBNP (per 1000 pg/mL increase) and hs-troponin at 48 h were strong independent predictors of in-hospital MACCE. These biomarkers capture the intensity of myocardial damage, wall stress, and neurohormonal activation, which are direct triggers for early complications.

While NT-proBNP and troponin remained independent predictors of MACCE in multivariable logistic regression, depression showed a borderline significant trend toward increased risk. This is consistent with prior studies showing that although depression influences in-hospital outcomes through behavioural and autonomic pathways, its effect is modest compared to that of acute myocardial injury during hospitalization. Myocardial infarction size, left ventricular dysfunction, and hemodynamic instability are primary drivers of MACCE, obscuring the influence of depression [27,59].

CRP also had a borderline statistical significance as an independent predictor of MACCE, while the interaction term depression x CRP did not reach statistical significance. However, this interaction analysis was exploratory and may have been underpowered because of the relatively small number of patients with depression and outcome events. Therefore, the absence of statistical significance should not be interpreted as definitive evidence that depression and inflammation act independently. Larger prospective studies are needed to further evaluate potential effect modification. These findings are consistent with prior studies demonstrating that depression and inflammation exert independent, additive effects on cardiovascular risk, with little evidence of effect modification between psychological and inflammatory pathways in the acute MI setting. Whooley et al. reported that in patients with stable coronary artery disease, inflammation is unlikely to account for the adverse cardiovascular outcomes associated with depression [60]. Vaccarino et al. found that although depression and inflammation were linked, both contributed to prognosis independently. Inflammation explained only a minor fraction of the increased cardiovascular risk observed in patients with depression [46]. Although much of the literature linking depression and inflammation to adverse cardiovascular outcomes originates from stable CAD cohorts, several studies conducted specifically in patients with AMI, including STEMI, support a similar pattern. Depressive symptoms have been associated with larger infarct size and greater myocardial injury on cardiac magnetic resonance imaging in patients with STEMI undergoing primary PCI, suggesting a heightened vulnerability to ischaemic damage in this group [61]. Other prospective STEMI cohorts have shown that depression is linked to a higher risk of in-hospital complications, including arrhythmias, acute HF, and hemodynamic instability [62].

An unexpected finding was the higher admission total cholesterol and LDL-cholesterol concentrations among patients who developed in-hospital MACCE. Admission lipid levels during acute STEMI may not accurately reflect chronic lipid status because they can be influenced by the acute-phase response, and information regarding pre-admission statin therapy was not consistently available. Importantly, neither total cholesterol nor LDL cholesterol remained independently associated with in-hospital MACCE after multivariable adjustment, suggesting that this univariable association was not independent.

Taken together, these findings suggest that in the acute STEMI setting, depression and inflammation may each contribute independently to early adverse outcomes, although the evidence base remains less extensive than in stable CAD. This underscores the importance and novelty of studies such as ours, which evaluate both psychological and inflammatory risk pathways within a high-risk STEMI population.

Most existing studies focus on short-term outcomes, but the long-term interaction between depression and inflammation is less explored. Because post-discharge complications are common and depression has long-term effects, we also assessed their impact on 12-month MACCE.

### 4.3. Depression, Inflammation and Long-Term MACCE

The independent association between depression and 12-month MACCE was evaluated while accounting for inflammation and cardiac functional parameters. The 12-month MACCE endpoint was reported in a total of 87 patients (29.8%) with excess risk in the subgroup with depression, 53.2% of whom had MACCE, more than double the number observed in patients without depression (23.5%, *p* < 0.001).

Lower 1-year MACCE rates have been reported in other studies; however, these findings are not directly comparable because of differences in composite endpoint definitions, particularly the exclusion of HF hospitalizations. For example, in the Western Denmark Heart Registry, which included 18,540 patients with first-time STEMI treated with primary PCI, the 1-year incidence of MACCE (defined strictly as recurrent MI, ischaemic stroke, and cardiovascular death) was 8.7%. The risk of recurrent MI between days 31 and 365 was 2.4% in 2015–2017, with ischaemic stroke at 1.5% and cardiovascular death at 5.3% [63].

Similar data were reported in our cohort, with 4.45% for the risk of recurrent MI (but significantly elevated in patients with depression (9.7% vs. 3.0%, *p* = 0.036)), 1.37% for the risk of stroke and 7.19% for cardiovascular death. Another study of patients with STEMI without cardiogenic shock at presentation who were treated with primary PCI reported a 1-year MACCE rate of 11.3%, defined as a composite of mortality, non-fatal recurrent MI, non-fatal stroke, and target vessel revascularization [64].

HF remains a common complication following STEMI despite advances in reperfusion therapy. HF develops in approximately 13% of patients within 30 days after MI and in 20–30% within the first year after discharge. Large national registry data from Norway, including over 86,000 first-time patients with AMI without prior HF, confirmed that HF frequently occurs both during the acute phase and early post-discharge period [65]. Similarly, data from the Harmonizing Outcomes With Revascularization and Stents in Acute Myocardial Infarction (HORIZONS-AMI) trial showed that the incidence of congestive HF increased from 2.6% before STEMI to 4.6% at 1 month and 4.7% at 1 year [66].

Our study found that 10.6% of patients with STEMI experienced severe HF requiring hospitalization within the first year after infarction, a figure that includes both in-hospital HF during the acute phase (Killip class III–IV) and hospitalizations for HF during follow-up. Notably, HF events were significantly more frequent among patients with baseline depression (22.6% vs. 7.4%, *p* = 0.002).

The primary endpoint was intentionally defined as cumulative MACCE from admission through 12 months to capture the overall burden of adverse cardiovascular events after STEMI. At 12 months, patients with depression at baseline had a significantly higher incidence of MACCE, driven primarily by an increased risk of recurrent MI and HF events. In multivariable analysis, depression remained an independent predictor of long-term MACCE. These findings highlight the sustained impact of psychological vulnerability on cardiovascular prognosis beyond the acute phase.

In Firth penalized logistic regression analysis, we identified four independent predictors of 12-month MACCE: depression and baseline SII, CRP, and LVEF at 12 months. These findings provide a coherent pathophysiological framework linking emotional, inflammatory, and cardiac remodelling factors to long-term cardiovascular risk [49,59]. Depression was a potent predictor even after adjusting for inflammation and ventricular function. The impact of depression on cardiovascular outcomes is independent, additive, and not mediated by interactions with inflammatory markers or LVEF.

This finding aligns with landmark post-MI studies demonstrating the prognostic relevance of depression that substantially increases cardiovascular mortality and recurrent events in patients with CAD. The ENRICHD trial follow-up showed that depression persisted as an independent predictor of mortality and recurrent MI even when behavioural and biological factors were considered [49]. Similarly, large post-MI cohorts and nationwide registry studies consistently report higher risks of mortality and recurrent cardiovascular events among patients with pre-existing depression even after adjusting for traditional risk factors and comorbidities [67,68].

Meta-analyses further estimate a 1.6–2.7-fold increased risk of adverse outcomes within 1–2 years post-MI among patients with depression. Although adjustment for disease severity attenuates the association, depression remains independently predictive, with each standard deviation increase in depressive symptom burden conferring incremental risk [69].

Thus, our findings reinforce depression as a high-impact prognostic marker and underline the need for psychosocial screening in cardiology.

Importantly, a landmark analysis restricted to patients discharged alive yielded similar findings, with pre-existing depression showing a borderline independent association with post-discharge MACCE. This suggests that the adverse prognostic impact of depression may extend beyond acute hospitalization and continue after discharge, although confirmation in larger prospective studies is warranted.

Our findings should be interpreted in the context of the depression phenotype evaluated. Because depression was defined using previously established psychiatric diagnoses, our results specifically reflect the prognostic impact of clinically diagnosed pre-existing depressive disorders rather than acute psychological distress or depression developing after STEMI. Previous studies suggest that incident post-MI depression may represent a partially distinct clinical phenotype, with different temporal characteristics, underlying biological mechanisms, and prognostic implications [10]. Therefore, our findings should not be extrapolated to patients who develop depressive symptoms only after the index infarction. Future prospective studies incorporating standardized depression assessments at admission and during follow-up are warranted to determine whether pre-existing and incident post-MI depression confer similar cardiovascular risk.

Both baseline SII and CRP independently predicted 12-month MACCE, underscoring the role of sustained immune-inflammatory activation in long-term cardiovascular deterioration. While baseline SII was not associated with in-hospital MACCE, likely because early complications are dominated by acute hemodynamic and procedural factors, it strongly predicted late events. This supports its role as an integrative marker of chronic inflammation, immune dysregulation, and prothrombotic activity contributing to residual cardiovascular risk after discharge. Consistent with our findings, other studies have identified the SII as a strong predictor of mortality and cardiovascular events in patients with ACS [42,70]. When ΔCRP was analysed instead of baseline CRP, it predicted outcomes in conventional logistic regression but lost significance in Firth penalized models. This discrepancy likely reflects small-sample bias or sparse data within CRP change strata, which Firth regression corrects. Moreover, ΔCRP may be influenced by treatment effects, regression to the mean, or intercurrent events before 12-month measurement, limiting its value as a stable prognostic marker. In contrast, baseline CRP captures the initial inflammatory burden during STEMI, which may exert lasting effects on myocardial injury, microvascular dysfunction, and vascular biology.

Notably, although inflammatory markers remained elevated at long-term follow-up among patients with depression in our cohort, the effect of depression on outcomes persisted independently of inflammatory dynamics. These findings suggest that depression represents a parallel vulnerability pathway rather than a purely inflammation-mediated mechanism.

This interpretation is consistent with evidence showing persistent low-grade inflammation in patients post-MI with depression [71,72] yet also with trials such as CANTOS demonstrating that targeted anti-inflammatory therapy reduces cardiovascular events independently of lipid levels [73], reinforcing the biological significance of inflammatory pathways. However, CANTOS enrolled clinically stable patients with a previous myocardial infarction receiving contemporary secondary prevention, including statin therapy, and therefore its findings cannot be directly extrapolated to the acute STEMI setting investigated in the present study.

### 4.4. Ventricular Recovery as a Determinant of Prognosis

LVEF at 12 months was a strong protective factor. This is expected, as ventricular remodelling and systolic recovery are key determinants of long-term prognosis after MI. The relationship between LVEF recovery, post-MI remodelling, and survival is well established in landmark post-MI trials and echocardiographic sub studies, with incremental improvements in LVEF associated with meaningful reductions in mortality [74,75]. In the COREA-AMI (Cardiovascular Risk and Identification of Potential High-Risk Population in Acute Myocardial Infarction) registry, HF with improved ejection fraction was associated with a significantly lower risk of all-cause mortality compared with HF without improvement in ejection fraction (HR 0.377; *p* < 0.001) [76].

Our data reaffirm that LVEF recovery remains a critical determinant of long-term prognosis even after adjusting for psychological and inflammatory factors.

We tested interactions between depression and SII, CRP, and 1 year-LVEF and found none. This suggests that depression does not amplify the detrimental effects of inflammation or poor EF. Thus, depression should be considered a distinct prognostic entity, not merely a modifier of biological risk pathways. The relationship between depression and cardiovascular disease is complex and influenced by multiple factors. Emerging research highlights mechanisms such as autonomic dysregulation and alterations in coagulation and hypothalamic–pituitary–adrenal axis activation, which may offer additional pathophysiological pathways connecting depression to CVD [28,77].

These findings underscore the multifaceted nature of cardiovascular risk in STEMI and support the need for integrated care strategies that address both biological and psychological domains. Depression screening, psychosocial support, and aggressive secondary prevention should be considered.

### 4.5. Study Limitations

Several limitations should be acknowledged. First, this was a retrospective, single-centre analysis, which may limit causal inference and generalizability of the findings to other populations and healthcare settings. Therefore, our results should be considered hypothesis-generating and require confirmation in larger prospective, multicentre studies. Depression was identified exclusively based on pre-existing psychiatric diagnoses documented before STEMI, and no standardized assessment of depressive symptoms (e.g., PHQ-9, BDI, or HADS) was available at the time of admission. Consequently, the overall burden of depression may have been underestimated, and the findings specifically reflect the prognostic impact of clinically diagnosed pre-existing depressive illness rather than the depressive symptom burden at STEMI presentation or reactive post-MI depression. Misclassification of patients with undiagnosed depression or subclinical depressive symptoms cannot therefore be excluded.

Second, information on antidepressant treatment, treatment adherence, lifestyle factors (including smoking cessation, physical activity, and cardiac rehabilitation), socioeconomic status, and psychosocial interventions during follow-up was not available due to the retrospective nature of the study. These factors may influence both depression status and cardiovascular prognosis and therefore could have acted as residual confounders. Future prospective studies should incorporate these variables to better characterize the relationship between depression and cardiovascular outcomes following STEMI. Third, although inflammatory markers were included, residual confounding related to unmeasured inflammatory or neurohormonal pathways cannot be excluded. The limited number of outcome events necessitated the use of Firth penalized regression; while this approach reduces small-sample bias and improves estimate stability, confidence intervals remain relatively wide, and findings should be interpreted accordingly.

Finally, ΔCRP was evaluated as a marker of inflammatory dynamics in sensitivity analyses, but serial inflammatory measurements beyond hospitalization were not available. Prospective studies with larger sample size, systematic psychological assessment and longitudinal biomarker profiling are warranted to further elucidate the mechanisms linking depression to adverse outcomes after STEMI.

Therefore, we focused on baseline depression diagnosis as a stable, categorical risk factor. While this may underestimate the dynamic influence of depression over time, the observed associations remain clinically relevant and highlight the prognostic importance of early psychological vulnerability in STEMI.

### 4.6. Clinical Implications

Our findings underscore the clinical importance of recognizing pre-existing depression as a marker of increased vulnerability in patients presenting with STEMI. Depression was independently associated with both in-hospital and 12-month MACCE even after adjustment for cardiac injury severity, ventricular function, and inflammatory markers. This suggests that depression represents a distinct and clinically relevant risk dimension that is not fully captured by traditional biological predictors.

Inflammatory biomarkers such as CRP and SII contributed to long-term risk stratification; however, the absence of significant interaction effects indicates that psychological and inflammatory processes likely operate in parallel rather than synergistically. Therefore, depression should not be viewed solely as an inflammatory surrogate but as an independent prognostic factor requiring direct clinical attention. Accurate early risk stratification remains essential in patients presenting with acute coronary syndrome. Several clinical risk scores have been developed to predict short-term adverse cardiovascular events after PCI [78]. Our findings suggest that incorporating psychological and inflammatory factors may provide complementary prognostic information beyond conventional clinical and laboratory parameters, although this hypothesis requires prospective validation.

From a practical standpoint, systematic identification of pre-existing depression at the time of STEMI admission may enhance risk stratification and support a more integrated, multidisciplinary approach to secondary prevention. Although our data do not establish causality or define specific therapeutic interventions, they reinforce the need for closer collaboration between cardiology and mental health services in the management of patients who are high risk post-MI.

## 5. Conclusions

Pre-existing depression was associated with significantly higher in-hospital and 12-month MACCE after STEMI, driven mainly by recurrent myocardial infarction and heart failure. Patients with depression exhibited a higher inflammatory burden, reflected by increased CRP and SII levels. The prognostic impact of depression remained independent and additive, without interaction with inflammatory or cardiac markers, highlighting depression as a clinically relevant and potentially modifiable condition for post-myocardial infarction risk stratification. Future prospective studies are needed to clarify the mechanisms linking depression to adverse outcomes in acute myocardial infarction and to determine whether targeted interventions addressing psychological distress may improve long-term prognosis in this high-risk population.

## Figures and Tables

**Table 1:** Baseline characteristics of patients with STEMI with and without depression.

Parameters	STEMI with Depression 62 Patients	STEMI Without Depression 230 Patients	*p*
Clinical data			
Age (years)	64.29 ± 11.71	64.94 ± 11.30	0.69
Sex F	37/62 (59.68%)	65/230 (28.26%)	<0.001
BMI (kg/m^2^)	27.69 ± 4.33	28.15 ± 4.60	0.508
Smoking	26/62 (41.94%)	110/230 (47.83%)	0.40
Hypertension	38/62 (61.29%)	136/230 (59.13%)	0.75
Diabetes	19/62 (30.65%)	71/230 (30.87%)	0.97
Length of hospital stay (days)	6.81 ± 2.36 days	5.32 ± 2.16	0.019
Laboratory data			
Total Cholesterol (mg/dL)	190.75 ± 45.00	188.13 ± 50.22	0.71
LDL Cholesterol (mg/dL)	126.85 ± 34.29	120.57 ± 40.30	0.26
HDL Cholesterol (mg/dL)	42.42 ± 40.11	38.68 ± 23.60	0.35
Triglycerides (mg/dL)	163.39 ± 105.45	166.21 ± 100.91	0.84
Hb (g/dL)	14.30 ± 1.61	14.53 ± 1.61	0.30
CRP (mg/L)	82.38 ± 63.30	47.42 ± 56.03	0.001
WBC (×10^3^/μL)	12.82 ± 5.45	12.14 ± 3.95	0.28
NLR	7.13 ± 4.7	4.84 ± 3.20	0.001
SII	1849.99 ± 1577.36	1303.70 ± 1003.65	0.012
Creatinine (mg/dL)	0.94 ± 0.35	0.96 ± 0.38	0.61
Hs-troponin admission (pg/mL)	6219.29 ± 10,261.61	10,179.88 ± 31,290.55	0.33
Hs-troponin 48 h (pg/mL)	42,436.12 ± 60,969.67	29,688.66 ± 33,474.12	0.035
NT-proBNP (pg/mL)	3326.41 ± 3894.05	4159.79 ± 6301.83	0.42
Imagistic data			
LVEF (%)	42.37 ± 8.77	42.21 ± 8.30	0.88
Coronary angiography	61/62 (98.39%)	228/230 (99.13%)	0.60
Number of affected vessels			
one	35/62 (56.45%)	125/230 (54.35%)	0.77
two	17/62 (27.41%)	51/230 (22.17%)	0.13
three	9/62 (14.52%)	52/230 (22.61%)	0.12
No reflow	19/62 (30.65%)	24/230 (10.43%)	<0.001

F, female; BMI, body mass index; LDL, low-density lipoprotein; HDL, high-density lipoprotein; Hb, haemoglobin; CRP, C-reactive protein; WBC, white blood cells; NLR, neutrophil-to-lymphocyte ratio; SII, systemic inflammatory index; Hs-troponin, high-sensitivity troponin; LVEF, left ventricular ejection fraction; NT-proBNP, N-terminal natriuretic peptide.

**Table 2:** In-hospital MACCE in patients with STEMI with and without depression.

In-Hospital Outcome	All Cohort	STEMI with Depression (62/292)	STEMI Without Depression (230/292)	*p*
MACCE	42/292 (14.38%)	15/62 (24.19%)	27/230 (11.74%)	0.014
All-cause death	11/292 (3.76%)	4/62 (6.45%)	7/230 (3.04%)	0.30
Cardiac death	10/292 (3.42%)	4/62 (6,45%)	6/230 (2.61%)	0.13
Resuscitated cardiac arrest	22/292 (7.53%)	7/62 (11.29%)	15/230 (6.52%)	0.21
Severe HF-Killip III. IV	24/292 (8.21%)	9/62 (14.52%)	15/230 (6.52%)	0.06
Recurrent MI	6/292 (2.05%)	4/62 (6.45%)	2/230 (0.87%)	0.03
Unplanned repeated revascularisation	5/292 (1.71%)	2/62 (3.23%)	3/230 (1.30%)	0.29
Stroke or TIA	3/292 (1.02%)	1/62 (1.61%)	2/230 (0.87%)	0.51

MACCE, major cardiac and cerebrovascular events; HF, heart failure; MI, myocardial infarction; TIA, transient ischaemic attack.

**Table 3:** Baseline characteristics of patients with STEMI with and without in-hospital MACCE.

Parameters	MACCE 42/292	No MACCE 250/292	*p*
Clinical data			
Age (years)	69.00 ± 10.93	64.12 ± 11.33	0.011
Sex F	16/42 (38.1%)	86/250 (34.4%)	0.65
BMI (kg/m^2^)	28.42 ± 4.54	28.05 ± 4.44	0.67
Smoking	16/42 (38.1%)	120/250 (48%)	0.24
Hypertension	17/42 (40.48%)	157/250 (62.8%)	0.007
Diabetes	16/42 (38.09%)	74/250 (29.6%)	0.28
Depression	15/42 (35.71%)	47/250 (18.8%)	0.02
Laboratory data			
Total Cholesterol (mg/dL)	193.38 ± 48.07	159.59 ± 46.28	<0.001
LDL Cholesterol (mg/dL)	126.12 ± 38.84	97.98 ± 31.84	<0.001
HDL Cholesterol (mg/dL)	34.29 ± 8.22	40.42 ± 30.11	0.009
Triglycerides (mg/dL)	157.79 ± 89.71	166.98 ± 103.74	0.60
Hb (g/dL)	14.13 ± 1.59	14.55 ± 1.61	0.12
CRP (mg/L)	87.13 ± 57.17	49.60 ± 58.06	<0.001
WBC (×10^3^/μL)	13.94 ± 6.30	12.03 ± 3.84	0.009
NLR	6.10 ± 4.48	5.23 ± 3.55	0.16
SII	1913.75 ± 1797.71	1343.22 ± 1014.56	0.004
Creatinine (mg/dL)	1.13 ± 0.46	0.92 ± 0.34	0.01
Hs-troponin admission (pg/mL)	11,667.64 ± 20,406.65	8996.03 ± 29,214.33	0.59
Hs-troponin 48 h (pg/mL)	51,534.31 ± 55,029.13	29,708.12 ± 38,537.18	0.003
NT-proBNP (pg/mL)	7821.98 ± 8778.76	3118.36 ± 4675.36	<0.001
Imagistic data			
LVEF (%)	40.14 ± 10.21	42.59 ± 7.98	0.08
Coronary angiography	41/42 (97.62%)	248/250 (99.2%)	0.37
Number of affected vessels one	20/42 (47.62%)	140/250 (56%)	0.27
two	11/42 (26.19%)	57/250 (22.8%)	0.56
three	10/42 (23.81%)	51/250 (20.4%)	0.65
No reflow	10/42 (23.8%)	33/250 (13.2%)	0.09

STEMI, ST-segment elevation myocardial infarction; F, female; BMI, body mass index; LDL, low-density lipoprotein; HDL, high-density lipoprotein; Hb, haemoglobin; CRP, C-reactive protein; WBC, white blood cells; NLR, neutrophil-to-lymphocyte ratio; SII, systemic inflammatory index; Hs-troponin, high-sensitivity troponin; LVEF, left ventricular ejection fraction; NT-proBNP, N-terminal natriuretic peptide.

**Table 4:** Multiple regression analysis for in-hospital MACCE independent predictors.

Parameters	*p*	OR	95% CI
Age (per 10 years)	0.665	1.12	0.70–1.76
Hs-troponin (48 h)_1000	0.035	1.008	1.001–1.015
NT-proBNP_1000	0.006	1.10	1.03–1.17
Depression	0.053	2.65	0.99–7.09
CRP (per 10 mg/L)	0.05	1.007	1.00–1.15

MACCE, major adverse cardiac and cerebrovascular events; Hs-troponin, high-sensitivity troponin; NT-proBNP, N-terminal pro-B-type natriuretic peptide; CRP, C-reactive protein; odds ratios (OR) with 95% confidence intervals (CI) are reported. Continuous variables are expressed per clinically meaningful increments (age per 10 years, CRP per 10 mg/L, NT-proBNP and hs-troponin per 1000 units).

**Table 5:** Twelve-month MACCE in patients with STEMI with and without depression.

12 Months	Entire Cohort	Depression 62/292 (21.23%)	No Depression 230 (78.76%)	*p*
MACCE	87/292 (29.79%)	33/62 (53.22%)	54/230 (23.48%)	<0.001
All-cause death	24/292 (8.22%)	7/62 (11.29%)	17/230 (7.39%)	0.308
Cardiac death	21/292 (7.19%)	6/62 (9.68%)	15/230 (6.52%)	0.18
Severe HF	31/292 (10.62%)	14/62 (22.58%)	17/230 (7.39%)	0.002
Recurrent MI	13/292 (4.45%)	6/62 (9.68%)	7/230 (3.04%)	0.036
Unplanned repeated revascularisation	26/292 (8.90%)	8/62 (12.90%)	18/230 (7.83%)	0.216
Stroke or TIA	4/292 (1.37%)	1/62 (1.61%)	3/230 (1.30%)	1.00

MACCE, major cardiac and cerebrovascular events; HF, heart failure; MI, myocardial infarction; TIA, transient ischaemic attack.

**Table 6:** Baseline parameters in patients with STEMI with and without 12-month MACCE.

Parameters at Baseline	MACCE 87/292 (29.79%)	No MACCE 205/292 (70.21%)	*p*
Depression	33/87 (37.93%)	29/205 (14.15%)	<0.001
Age (years)	65.20 ± 11.64	64.63 ± 11.28	0.699
CRP (mg/L)	76.86 ± 63.20	47.55 ± 58.02	<0.001
NT-proBNP (pg/mL)	6076.76 ± 7176.97	2901.46 ± 4677.96	0.004
Hs-troponin 48 h (pg/mL)	44,251.28 ± 53,076.83	27,850.66 ± 35,085.69	0.003
SII	1997.64 ± 1164.07	1171.52 ± 799.25	<0.001
Diabetes	27/87 (31.03%)	63/235 (26.80%)	0.532
Hypertension	48/87 (55.17%)	126/205 (61.46%)	0.15
Total Cholesterol (mg/dL)	178.17 ± 49.57	191.33 ± 48.81	0.894
LDL cholesterol (mg/dL)	114.05 ± 42.08	123.79 ± 38.29	0.901
Triglycerides (mg/dL)	156.29 ± 87.15	168.02 ± 105.16	0.193
HDL cholesterol (mg/dL)	36.59 ± 10.93	40.73 ± 32.64	0.258
LVEF (%)	39.16 ± 9.87	43.59 ± 7.24	<0.001

STEMI, ST-segment elevation myocardial infarction; LDL, low-density lipoprotein; HDL, high-density lipoprotein; CRP, C-reactive protein; SII, systemic inflammatory index; Hs-troponin, high-sensitivity troponin; LVEF, left ventricular ejection fraction; NT-proBNP, N-terminal natriuretic peptide.

**Table 7:** Twelve-month parameters in patients with STEMI with and without MACCE.

Parameters	MACCE 87/292 (29.79%)	No MACCE 205/292 (70.21%)	*p*
CRP (mg/L)	42.33 ± 4.02	4.02 ± 4.61	0.001
ΔCRP (mg/L)	−33.99 ± 85.95	−40.56 ± 56.24	0.001
NT-proBNP (pg/mL)	2732.937 ± 5926.150	2239.9229 ± 5713.3346	0.828
ΔNT-proBNP (pg/mL)	−870.169 ± 7865.13	−2153.92 ± 4616.88	0.225
SII	1956.9845 ± 944.36778	468.897 ± 832.65927	<0.001
ΔSII	−641.17 ± 1741.98	−870.17 ± 1298.99	0.63
LVEF (%)	39.11 ± 11.23	47.99 ± 7.24	<0.001
ΔLVEF (%)	1.28 ± 9.65	5.10 ± 8.37	0.026
Diabetes	28/87 (32.18%)	65/205 (31.70%)	0.49
Total cholesterol (mg/dL)	137.46 ± 31.04	125.85 ± 49.65	0.30
LDL cholesterol (mg/dL)	74.87 ± 25.75	68.67 ± 35.53	0.34
HDL cholesterol (mg/dL)	33.71 ± 8.09	40.01 ± 9.65	0.001
Triglycerides (mg/dL)	123.38 ± 62.201	101.205 ± 40.76	0.046

STEMI, ST-segment elevation myocardial infarction; LDL, low-density lipoprotein; HDL, high-density lipoprotein; CRP, C-reactive protein; SII, systemic inflammatory index; LVEF, left ventricular ejection fraction; NT-proBNP, N-terminal natriuretic peptide; Δ-absolute change between baseline and at 12 months.

**Table 8:** Multivariable Firth penalized logistic regression analysis identifying independent predictors of 12-month MACCE.

Parameter	*p*	OR	95% CI	
Depression	0.045	3.35	1.03	11.34
Baseline SII_1000	<0.001	4.89	2.04	13.58
12-month LVEF (per 5%)	0.019	0.69	0.5	0.94
Baseline CRP (per 10 mg/L)	0.003	1.48	1.11	2.61

MACCE, major adverse cardiac and cerebrovascular events; SII, systemic immune-inflammation index; LVEF, left ventricular ejection fraction; CRP, C-reactive protein. Odds ratios (OR) with 95% confidence intervals (CI) are reported. Continuous variables are expressed per clinically meaningful increments.

## Data Availability

The original contributions presented in this study are included in the article. Further inquiries can be directed to the corresponding authors.

## Abbreviations

The following abbreviations are used in this manuscript:

ACE	Angiotensin-converting enzyme
ACS	Acute coronary syndrome
AMI	Acute myocardial infarction
BMI	Body mass index
CAG	Coronary angiography
CAD	Coronary artery disease
CBC	Complete blood count
CI	Confidence interval
COREA-AMI	Cardiovascular Risk and Identification of Potential High-Risk Population in Acute Myocardial Infarction
CRP	C-reactive protein
CT	Computed tomography
CVD	Cardiovascular disease
DEPACS	Depression in Acute Coronary Syndrome
ECG	Electrocardiogram
EF	Ejection fraction
ENRICHD	Enhancing Recovery in Coronary Heart Disease
ESC	European Society of Cardiology
Hb	Haemoglobin
HDL	High-density lipoprotein
HF	Heart failure
HORIZONS-AMI	Harmonizing Outcomes with Revascularization and Stents in Acute Myocardial Infarction
HR	Hazard ratio
hs-TnI/Hs-TnI	High-sensitivity troponin I
IBM	International Business Machines
LDL	Low-density lipoprotein
LVEF	Left ventricular ejection fraction
MACCE	Major adverse cardiovascular and cerebrovascular events
MBG	Myocardial blush grade
MI	Myocardial infarction
MRI	Magnetic resonance imaging
NLR	Neutrophil-to-lymphocyte ratio
NT-proBNP/NT proBNP	N-terminal pro-B-type natriuretic peptide
OR	Odds ratio
PCI	Percutaneous coronary intervention
P2Y12	Purinergic receptor P2Y12
SII	Systemic immune-inflammation index/Systemic inflammatory index
SPSS	Statistical Package for the Social Sciences
STEMI	ST-segment elevation myocardial infarction
TIA	Transient ischaemic attack
TIMI	Thrombolysis in Myocardial Infarction
WBC	White blood cells
